# An in vitro study on the effects of serum proteins on *Enterococcus faecalis* adhesion to three types of root sealers and gutta-percha

**DOI:** 10.1186/s12903-021-01992-4

**Published:** 2021-12-07

**Authors:** Xinwei Lin, Danlu Chi, Qimei Gong, Zhongchun Tong

**Affiliations:** 1 Department of Operative Dentistry and Endodontics, Guanghua School of Stomatology, Sun Yat-Sen University, No 56, Lingyuan West Road, Guangzhou, 510055 Guangdong China; 2 Guangdong Provincial Key Laboratory of Stomatology, Sun Yat-Sen University, Guangzhou, Guangdong China

**Keywords:** *Enterococcus faecalis*, Root canal sealers, *Fetal bovine serum*

## Abstract

**Background:**

The extrusion of overfilled materials that extend beyond the apical foramina into the periradicular tissue may serve as a reservoir for bacterial adhesion and further affect recovery from periapical diseases. The aim of this study was to evaluate the effects of serum proteins on *Enterococcus faecalis* adhesion and survival on the surface of a calcium hydroxide-based root canal sealer (Apexit Plus), an epoxy resin sealer (AH-Plus) and a bioceramic sealer (iRoot SP).

**Methods:**

Apexit Plus, AH-Plus and iRoot SP were evenly coated on gutta-percha, using gutta-percha alone as the control. After root canal sealer setting, the number of *E. faecalis* adhering to the root canal sealers and gutta-percha was counted in fetal bovine serum (FBS) or tryptic soy broth supplemented with 1% glucose (TSBG) by viable cell plate counts. The morphology of 7-day-old *E. faecalis* biofilms in FSB and TSBG was observed by scanning electron microscopy (SEM). Furthermore, *E. faecalis* biofilms on the three root canal sealers were labeled with a LIVE/DEAD BacLight™ Bacterial Viability Kit, and the ratios of viable to dead cells were analyzed using laser scanning microscopy operative software (Zen software).

**Results:**

In the assays, after 1 and 7 days, the number of *E. faecalis* adhering to the root canal sealers or gutta-percha in FBS were significantly lower than those in TSBG (*P* < 0.05). In FBS, *E. faecalis* adhesion to iRoot SP and gutta-percha was reduced to a greater extent than that adhered to Apexit Plus and AH-Plus. Few *E. faecalis* accumulated on iRoot SP in FBS, whereas many bacteria assembled on iRoot SP and formed biofilms in TSBG. The ratio of viable cells in the *E. faecalis* biofilm on iRoot SP was the lowest.

**Conclusions:**

Calcium hydroxide-based root canal sealers, epoxy resin sealers and bioceramic sealers may provide a substrate for *E. faecalis* adhesion, and the bioceramic sealer in this study showed the least *E. faecalis* adhesion in the presence of serum proteins compared to the other two sealers.

## Background

Adequate root canal obturation prevents bacterial invasion of the root canal space and is one of the key factors in successful endodontic treatment [1]. Although chemomechanical preparation and medicaments may remove most of the pathogenic bacteria from the root canal system, some areas, such as isthmuses, lateral canals, accessory canals, and other root canal irregularities, remain untouched [2]. These untouched areas with residual bacteria will prevent the healing of periradicular lesions.

During root canal obturation, root canal sealers may entomb residual bacteria and act as disinfectants to prevent bacterial invasion [3, 4]. Among the current root canal sealers, the calcium hydroxide-based root canal sealer Apexit Plus and the tricalcium silicate-based bioceramic sealer iRoot SP have shown strong antimicrobial activities and excellent physical and biological properties; therefore, they have been widely applied for root canal obturation. An epoxy resin-based sealer, such as AH Plus, is generally accepted to be the gold standard against all new sealers due to its resorption resistance and dimensional stability [5–10]. Endodontic sealers possess antimicrobial activity against a range of common pathogenic microbes and provide a complete microscopic seal to impede bacterial invasion of the root canal system [3, 11–13]. However, gutta-percha points enclosed by endodontic sealers may still provide a substrate for bacterial adhesion despite their antibacterial activities. The extraradicular presence of the filling material may delay the healing of periradicular diseases or even cause treatment failure [14–16].

Gutta-percha and sealers are generally well tolerated on apical tissues in the absence of concomitant infection, and even in the presence of filling material extrusion, histological healing may still occur [17, 18]. However, in the presence of extrusion of infected debris by overinstrumentation or infection in the periradicular tissues, the extruded gutta-percha and root canal sealers may serve as a biofilm reservoir for sustained infection of the periradicular area, which might contribute to a nonhealing outcome [19]. *Enterococcus faecalis* species are considered to be the predominant bacteria in root-filled teeth with therapy failure. Some studies have found *E. faecalis* and bacterial biofilms on gutta-percha filling materials obtained from teeth associated with refractory radicular periodontitis [5, 20, 21]. At present, studies regarding bacterial adhesion to root canal sealers as well as the antibacterial activities of these sealers have been evaluated in a common bacterial culture medium [5–7]. However, in vivo, the extruded gutta-percha and root canal sealer are located at the periradicular tissue. Therefore, to elucidate bacterial adhesion after material overfilling to the periradicular tissue, we need to investigate the effects of serum proteins on *E. faecalis* biofilm adhesion to the surface of gutta-percha coated with three common root canal sealers, Apexit Plus, AH-Plus and iRoot SP sealer.

## Methods

### Bacterial culture

*Enterococcus faecalis* OG1RF was selected as the test bacteria in this study. A culture of the bacteria from the freezer (-80 °C) was streaked onto brain–heart infusion agar (BHI; Difco Laboratories, Detroit, MI) plates and grown at 37 °C for 24 h. A single colony of bacteria was inoculated into 5 mL of BHI broth and cultured at 37 °C under anaerobic conditions until the exponential phase of growth.

### Gutta-percha coating by the root canal sealers

Three resin blocks with simulated curved root canals (Plastic Training Block V04 0245; VDW, Munich, Germany) were instrumented using a ProTaper system (Dentsply Maillefer, Ballaigues, Switzerland) up to F2 accompanied by irrigation with 3% sodium hypochlorite (NaOCl) and 17% ethylenediaminetetraacetic acid (EDTA). The three prepared root canals were filled with 5.25% NaOCl and disinfected for 10 min. After being drying with sterile paper points, the three prepared root canals were filled with one of three root canal sealers: Apexit Plus (a calcium hydroxide-based root canal sealer, Ivoclar Vivadent, Liechtenstein), AH-Plus (Dentsply De Trey GmbH, Konstanz, Germany) or iRoot SP (a tricalcium silicate–containing sealer, Innovative BioCeramix Inc., Vancouver, BC, Canada). After sterilization by soaking in 5.25% NaOCl for 5 min, nine ProTaper F2 gutta-percha points were inserted into the three groups of root canals filled with sealer and rotated back and forth 360° to ensure that the root canal sealer was evenly coated on the surface of the gutta-percha. After solidification, the gutta-percha with root canal sealer and the control gutta-percha without sealer were cut off at the 10 mm site from the tip and placed in a 24-well microplate.

### Evaluation of bacterial adhesion

To evaluate the effects of the root canal sealers on bacterial adhesion, 1.99 mL of tryptic soy broth supplemented with 1% glucose (TSBG) and 10 µL of *E. faecalis* in the exponential growth phase were added to a 24-well microplate and incubated at 37 °C for up to 1 day or 7 days. The culture was replaced with fresh medium every second day. After incubation, the four groups of gutta-percha points were removed and washed two times with sterile phosphate-buffered saline (PBS) solution to remove the unadhered cells. The gutta-percha points were then placed in a centrifuge tube containing 2 mL of sterile PBS and stored in an ice box. The adhesive bacteria were harvested by scraping and ultrasonic treatment and resuspended in PBS. Afterward, the bacterial suspension was consecutively diluted tenfold and spread on BHI agar plates. The colonies were counted, and the number of bacteria adhered to the four groups of gutta-percha was calculated. To evaluate the effects of serum proteins on bacterial adhesion, the same four groups of gutta-percha were immersed in 1.99 mL of fetal bovine serum (FBS; HyClone, America) and 10 µL of *E. faecalis* in the exponential growth phase, and the same bacterial adhesive assay was performed. Each test was carried out at least 3 times on different days.

### Scanning electron microscopy

The three groups of gutta-percha points with root canal sealers and a control gutta-percha point without sealer were prepared, and 7 days of *E. faecalis* adhesion in TSBG or FBS was completed according to the above procedure. The gutta-percha specimens were fixed in 2.5% glutaraldehyde and dehydrated in a series of acetonitrile solutions (50%, 70%, 80%, and 90% for 20 min each and 100% for 20 min twice) after being rinsed twice with sterile PBS. The specimens were dried and then sputtered with gold. *E. faecalis* biofilms on the surface of the gutta-percha points were observed by scanning electron microscopy (SEM; E-1010, Hitachi, Ibaraki, Japan).

### Confocal laser scanning microscopy

The Apexit Plus, AH-Plus and iRoot SP sealers were evenly coated on the surface of a 10-mm diameter cover glass. After solidification at 37 °C, the cover glasses were immersed in FBS for 2 h in a 24-well microplate. The cover glasses without sealers were referred to as the control. The four groups of cover glasses were transferred to another well, and 1.99 mL of TSBG and 10 µL of *E. faecalis* in the exponential growth phase were added. After incubation at 37 °C for 7 days, the bacterial biofilms on the surfaces of the root canal sealers were gently washed twice with PBS. Subsequently, the cover glasses with bacterial biofilms were transferred to Petri dishes with glass bottoms (D: 35 mm, Hangzhou Shengyou Biotechnology, China) and stained with SYTO 9 and propidium iodide (PI) at room temperature in the dark for 15 min according to the specifications of the L7012 kit (LIVE/DEAD Bac-Light Bacterial Viability Kit; Molecular Probes, Eugene, OR). The Petri dishes were then clamped, and bacterial biofilms were scanned with a Carl Zeiss confocal laser scanning microscopy (CLSM) instrument. SYTO 9 and PI were excited at 488 nm and 543 nm, respectively. Three-dimensional biofilms were captured along the Z axis, and the ratio of viable and dead cells in each biofilm was calculated by ZEN software (ZEN 2012 light edition, Carl Zeiss MicroImaging, Inc., Thornwood, NY), which is a modular image acquisition, processing and analysis software for digital microscopy.

### Statistical analysis

Statistical analysis was performed using SPSS 20.0 software. The number of *E. faecalis* adhesions on the surfaces of the three root canal sealers and the control group were compared using one-way ANOVA and Tukey’s HSD tests. A *P* value < 0.05 was considered statistically significant.

## Results

### Effects of the sealers on bacterial adhesion

**Fig. 1:**
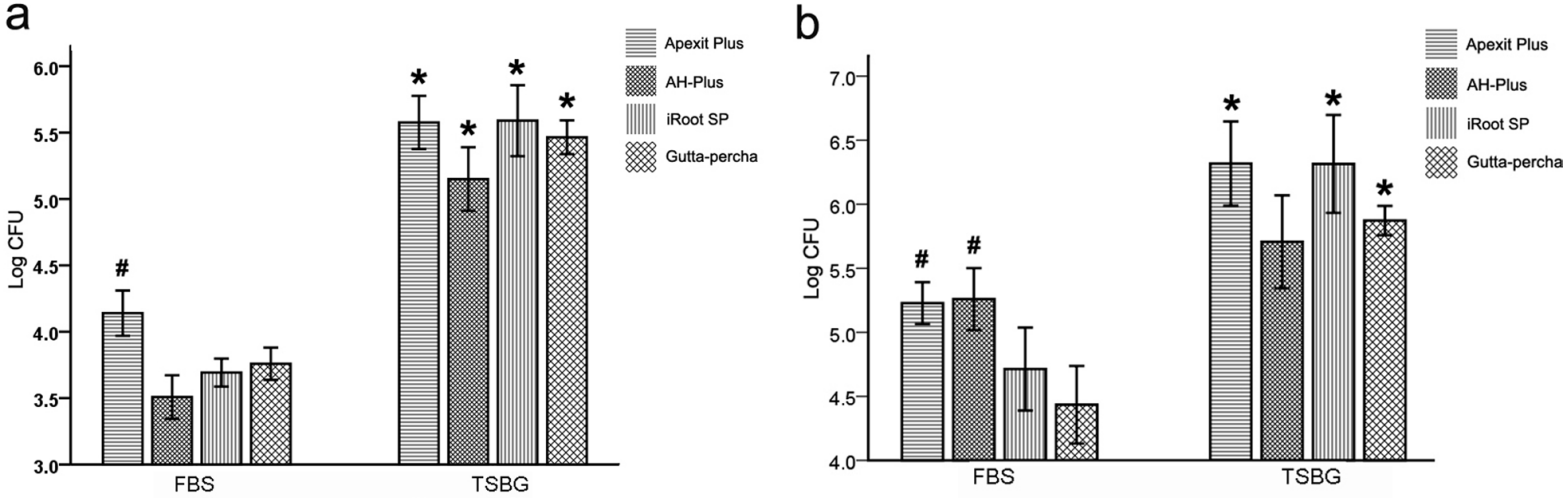
The amount of *E. faecalis* adhering to Apexit Plus, AH-Plus, iRoot SP and gutta-percha alone in FBS or TSBG medium for 1 day (**a**) and 7 days (**b**). ‘‘*’’ represents a significant difference between the amount of *E. faecalis* adhered to the same sealer in FBS and TSBG media (*P* < 0.05). ‘‘#’’ represents a significant difference compared to the amount of *E. faecalis* adhered to gutta-percha alone in the same medium (*P* < 0.05)

On both Days 1 (Fig. 1a) and 7 (Fig. 1b), the amount of *E. faecalis* adhering to Apexit Plus, iRoot SP and gutta-percha in TSBG was significantly greater than that in FBS (*P* < 0.05), and there was no significant difference between the amount of *E. faecalis* adhering to AH-Plus in TSBG and FBS on Day 7 (*P* > 0.05). *E. faecalis* adhesion to Apexit Plus was significantly greater than that to iRoot SP and gutta-percha in FBS (*P* < 0.05) on Days 1 and 7. *E. faecalis* adhesion to AH-Plus was significantly greater than that to the iRoot SP and gutta-percha in FBS only on Day 7. The four groups exhibited no significant difference in TSBG (*P* > 0.05). Whether in FBS or in TSBG, of the amount of *E. faecalis* adhesion after 7 days was greater than that after 1 day of adhesion in the four groups (Fig. 1).

### Morphology of bacterial biofilm adhesion to the surface of the sealers and gutta-percha

**Fig. 2:**
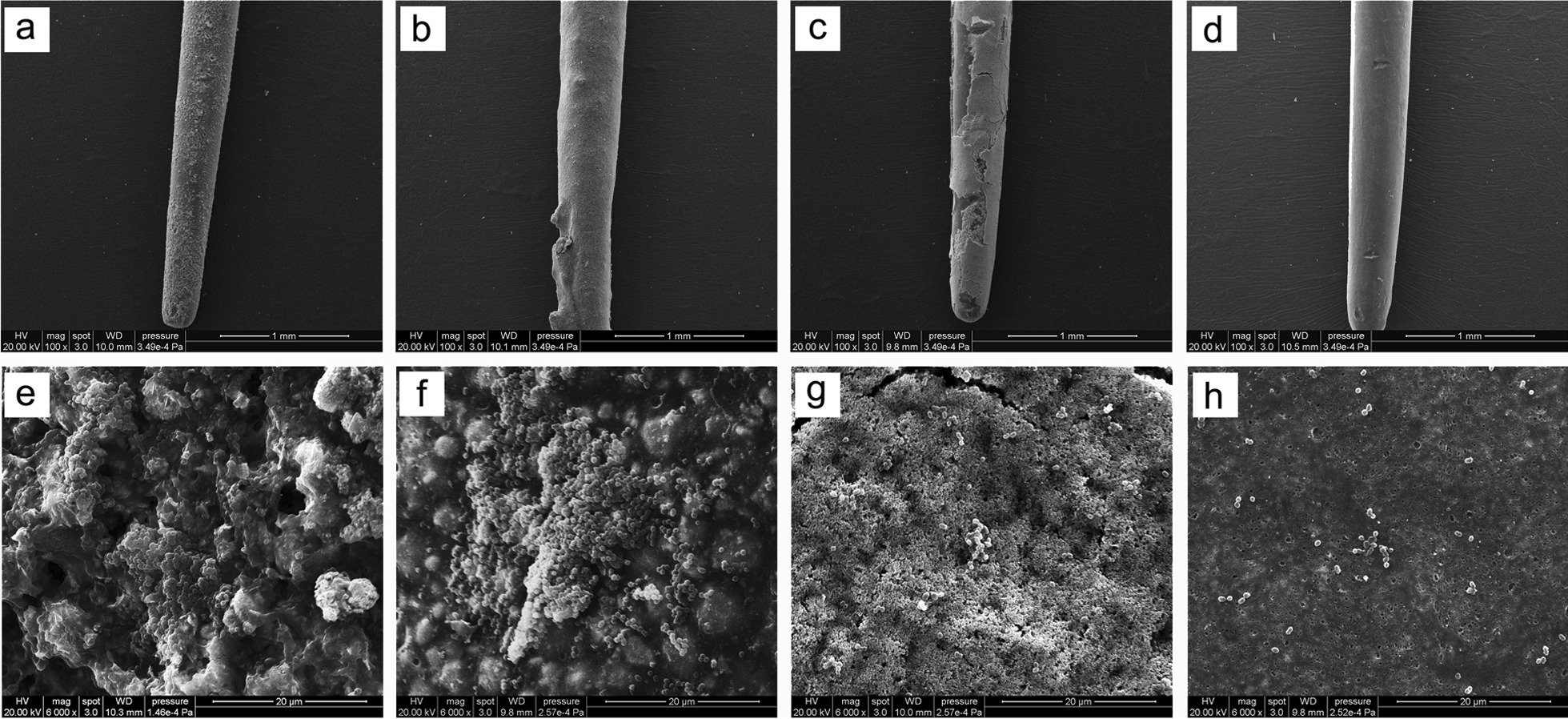
*E. faecalis* adhesion to Apexit Plus (**a**) and (**e**), AH-Plus (**b**) and (**f**), iRoot SP (**c**) and (**g**), and gutta-percha points (**d**) and (**h**) in FBS medium. **a**–**d** ×100 magnification and **e**–**h** show ×6000 magnification

**Fig. 3:**
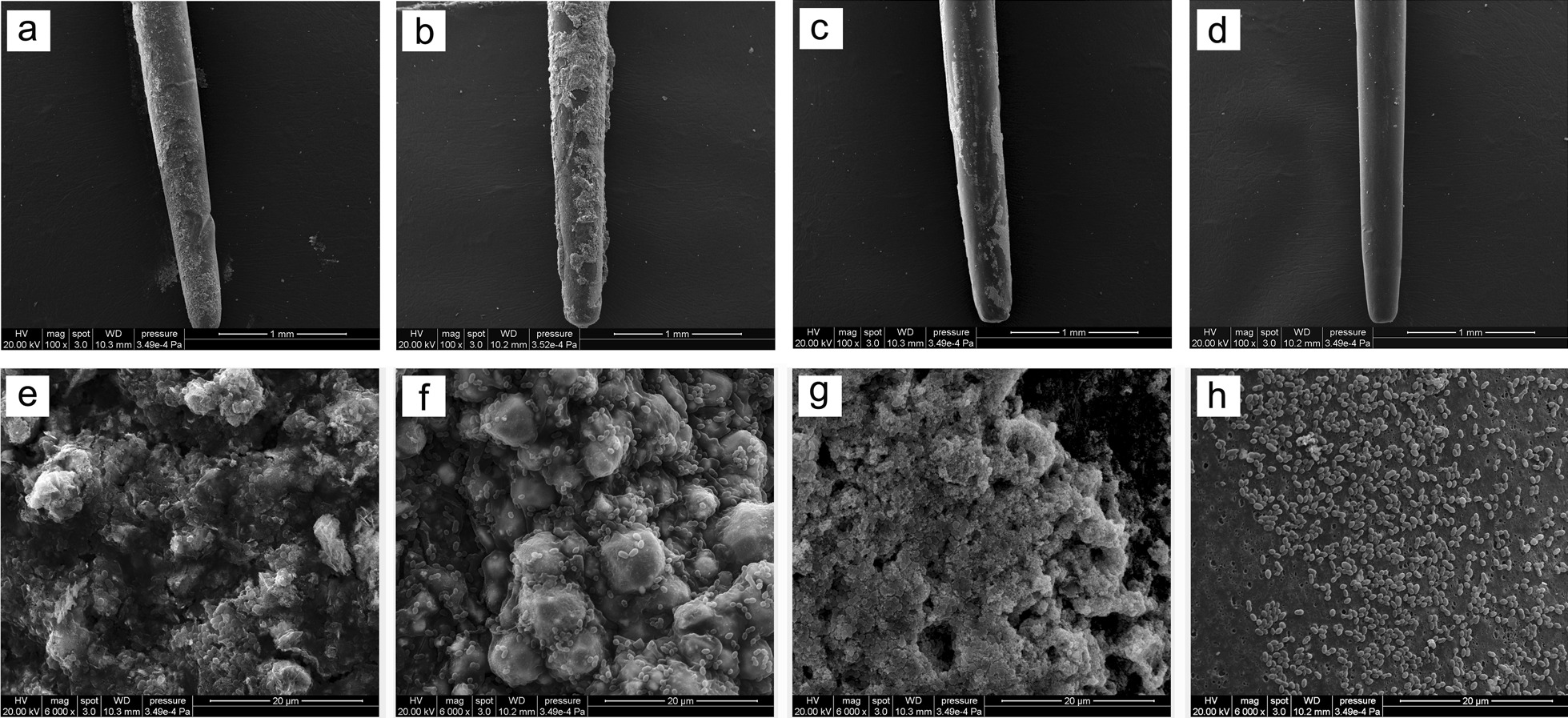
*E. faecalis* adhesion to the surfaces of Apexit Plus (**a**) and (**e**), AH-Plus (**b**) and (**f**), iRoot SP (**c**) and (**g**), and gutta-percha points (**d**) and (**h**) in TSBG medium. **a**–**d** ×100 magnification and **e**–**h** show ×6000 magnification

Gutta-percha coated with three root canal sealers and the control gutta-percha without sealer were observed by SEM after being immersed in FBS and TSBG medium for 7 days. Few bacteria adhered to iRoot SP and gutta-percha, and the masses of the bacteria aggregated on Apexit Plus and AH-Plus in FBS medium (Fig. 2). In TSBG medium, a number of *E. faecalis* aggregated on Apexit Plus and iRoot SP and showed a typical biofilm structure (Fig. 3e, g). A small amount of relatively loose *E. faecalis* adhered to AH-Plus (Fig. 3f). Furthermore, a few *E. faecalis* were dispersedly adhered to the gutta-percha and did not form a biofilm structure (Fig. 3h).

### Antibacterial activities of the three root sealers on *Enterococcus faecalis* biofilms

**Fig. 4:**
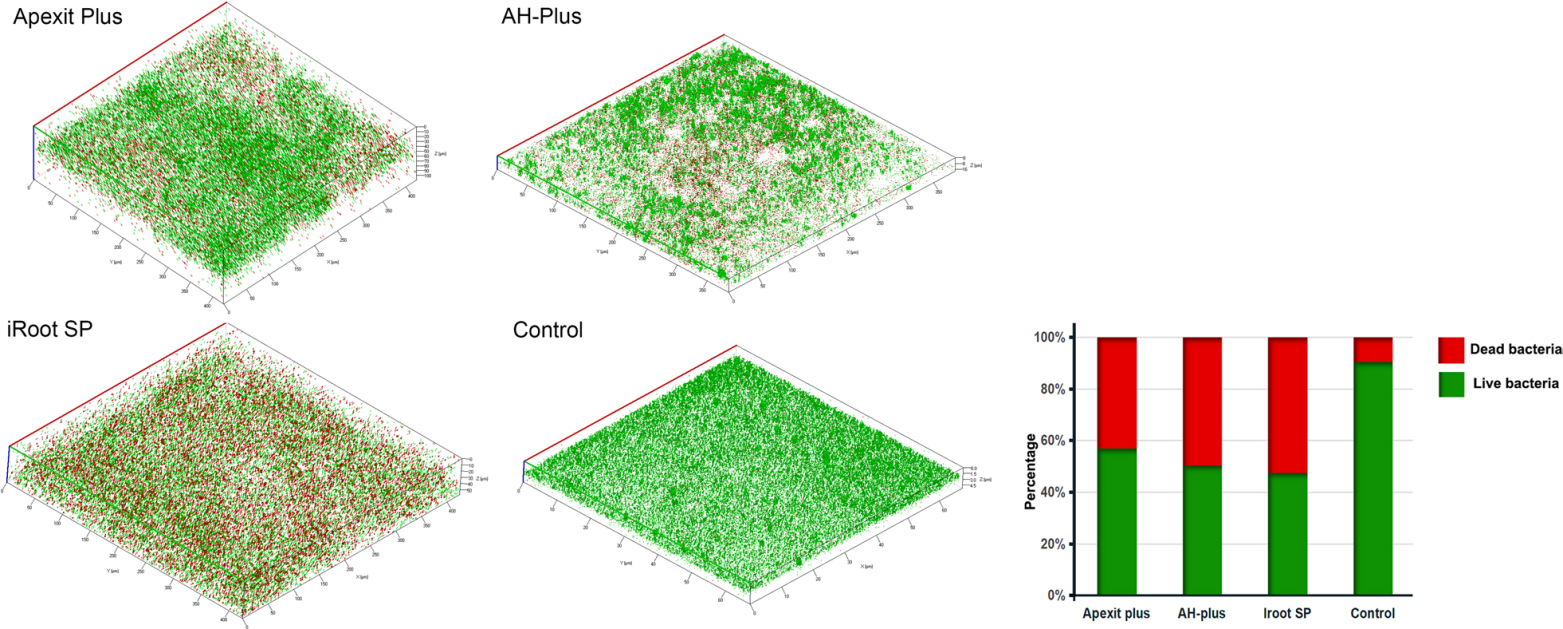
CLSM images of 3-dimensional *E. faecalis* biofilms on the surfaces of Apexit Plus, AH-Plus, iRoot SP and the control without sealer. Dead cells in biofilms are stained red, while live cells are stained green using the LIVE/DEAD Bac-Light Bacterial Viability L7012 Kit. The percentages of live and dead bacteria in the 3-dimensional *E. faecalis* biofilms were calculated by ZEN 2012 software

Images of 3-dimensional *E. faecalis* biofilms on the surfaces of the root canal sealers are shown in Fig. 4. For the control, *E. faecalis* biofilms mostly showed green live bacteria. Red dead bacteria were found on the surface of the three root canal sealers, and the percentages of dead bacteria on the iRoot SP sealer were the highest among the three groups of root canal sealers.

## Discussion

Filling materials should be restricted to the intraradicular space during root canal obturation, and endodontic treatment with root canal fillings ending within 2 mm of the scope of the radiographic apex show a high success rate [22]. However, the extrusion of root canal filling materials occasionally occurs due to incorrect operations, anatomical factors, and material properties, among other factors. Extruded filling materials may affect the treatment outcome, and a significantly worse outcome has been observed for teeth with apical periodontitis in comparison with teeth with no lesions [17]. Extrusion not only causes an inflammatory response but also provides a substrate for bacterial adhesion [23, 24]. Although filling root canal sealers have antibacterial activities, some bacteria, such as *E. faecalis,* still adhere to the surface of the materials [5, 25]. Our studies indicated that *E. faecalis* may adhere to the surface of Apexit Plus, AH-Plus, iRoot SP, and gutta-percha. Notably, substrate of overfilling might be conducive to the formation of bacterial biofilms and further hinder the healing of apical periodontitis.

The antibacterial activities of root canal sealers against *E. faecalis* have mostly been measured by bacterial growth in common medium [13, 26–28]. In these tests, the root canal sealers showed different antibacterial activities. A study by Bukhari et al. showed that Endosequence BC (which is the same sealer as iRoot SP marketed under a different brand name) killed significantly more 8-week-old *E. faecalis* in biofilms attached to the canal surfaces when compared with AH-Plus by confocal laser scanning microscopy analysis [13]. Candeiro et al. showed no bacterial growth from 24 to 168 h in the medium after the insertion of AH-Plus or BC sealer by using a direct contact test (DCT) [23]. To determine the antibacterial activities of root canal sealers, DCTs are often employed. However, when the root canal sealers or gutta-percha are overfilled in the periradicular tissues, the amount of bacteria adhered to the root canal sealers cannot be tested by DCT only due to the existence of tissue fluid. The effects of the proteins in tissue fluid on the antibacterial activities of the tested drugs was evaluated by using FBS, human serum and BSA [29–31]. When extruded materials exist in periradicular tissues, the antibacterial action of the root canal sealers may not be the same as those obtained from in vitro tests in bacterial medium. Our studies showed that the amount of *E. faecalis* adhering to the three root canal sealers and gutta-percha was lower in FBS than in TSB. Root canal sealer dissolves as a result of contact with serum-like fluid or bacterial medium, and interstices are made, which allows bacterial adhesion. Epoxy resin sealers are generally considered to be difficult to dissolve [9, 10], and thus, there was no significant difference between the amount of *E. faecalis* adhesion to AH-Plus in FBS or TSB at 7 days. However, calcium hydroxide-based root canal sealers and bioceramic sealers may dissolve slightly and produce greater porosities [32, 33]. TSBG is a nutrient-rich medium that includes many proteins and carbohydrates, and it has benefits against bacterial growth and adhesion. Serum proteins mostly provide an organic monolayer film on root canal sealers to serve as an excellent anchor for bacterial adhesion. Calcium hydroxide-based root canal sealers and bioceramic sealers are more strongly dissolved in TBS because FBS is a slightly viscous liquid. Thus, more *E. faecalis* adhered to Apexit Plus and iRoot SP in TSBG than in FBS.

The amount of *E. faecalis* adhering to AH-Plus significantly decreased in FBS on day one, and the bacterial amount increased on Day 7, which was consistent with the Kapralos et al. study. *E. faecalis* tested on the surface of the root canal sealers coated on gutta-percha in our studies and *E. faecalis* counts by a modified DCT assay in the Kapralos et al. study both showed that AH Plus had high antibacterial activity on day one, and the antibacterial activity decreased over 7 days despite the different test methods [11]. The amount of viable *E. faecalis* was reduced on the surface of iRoot SP compared to that of AH-Plus on Day 7, which indicated that the bioceramic sealers showed durable antibacterial activities against *E. faecalis*. Freshly mixed root canal sealers are often considered to have certain antimicrobial activity against *E. faecalis*, although the antimicrobial activity was lost as the material set with the exception of the bioceramic sealers [34].

Gutta-percha is the most popular core material used for root canal obturation. In our study, few *E. faecalis* adhered to the gutta-percha without sealers in the presence of FBS, and the amount of bacterial adhesion after 7 days was the least in the four test groups on gutta-percha alone. The relatively smooth surface of gutta-percha did not benefit bacterial adhesion. Gutta-percha cones consist of approximately 20% gutta-percha, 65% zinc oxide, 10% radiopacifiers, and 5% plasticizers, and attempts have been made to make gutta-percha antimicrobial by the addition of antibacterial materials [35–38], which will further decrease bacterial adhesion. In George et al.’s studies, when *E. faecalis* 29212 was inoculated, no significant biofilm formation was detected at the gutta-percha points conditioned with serum for 2 weeks under nutrient-deprived conditions, similar to our results [39]. However, in the study by Takemura et al., a significant bacterial biofilm formed on the gutta-percha points when *E. faecalis* 6-2L was used as the test bacteria in media containing 45% and 90% serum [40]. These different results might be related to the pretreatment of the gutta-percha points. The gutta-percha points were sterilized by soaking in 5.25% NaOCl for 5 min before evaluation in our study, and pretreatment with NaOCl significantly reduced *E. faecalis* formation on gutta-percha points [41]. Therefore, bacterial adhesion to the gutta-percha points was influenced by multiple factors, such as the bacterial strains, nutrition, disinfectants, and infection time.

An in vitro study investigated the effects of serum proteins on *E. faecalis* adhesion to the surface of three root canal sealers coated on gutta-percha. The extrusion of root canal sealers or gutta-percha in periradicular tissues initiates inflammatory reactions [42, 43]. The immune response by the overfilled materials and bacterial clearance of the immune system also influence bacterial adhesion to the sealers and gutta-percha. Therefore, future studies on the effects of bacterial adhesion to the extruded materials on periapical tissue should be considered in combination with serum proteins and host immune cells.

## Conclusion

Within the limitations of this study, despite the antibacterial action of a calcium hydroxide-based root canal sealer, an epoxy resin sealer and a bioceramic sealer, *E. faecalis* may still adhere to the surface of these three root canal sealers coated on gutta-percha. Serum proteins significantly reduced the amount of *E. faecalis* adhered to the three types of root canal sealers, and *E. faecalis* showed less adhesion to the bioceramic sealer and gutta-percha alone than the calcium hydroxide-based root canal sealer and epoxy resin sealer in the presence of serum proteins.

## Data Availability

All data generated or analyzed during this study are included in this published article.

## Abbreviations

FBS: Fetal bovine serum
TSBG: Tryptic soy broth supplemented with 1% glucose
NaOCl: Sodium hypochlorite
SEM: Scanning electron microscopy
CLSM: Confocal laser scanning microscopy
